# Supplementary material for: Trends in Beef Intake in the United States: Analysis of the National Health and Nutrition Examination Survey, 2001–2018
Source: Nutrients. 2023 May 26;15(11):2475. doi: 10.3390/nu15112475 (PMC10255758; doi:10.3390/nu15112475)
Supplement: Supplementary file 1 [file nutrients-15-02475-s001.zip › nutrients-2356751-supplementary.pdf]

Figure S1. Determination of Red Meat Contribution to Healthy U.S. Style Eating Pattern (HDP) at the 2,000-calorie level

Step 1. Determine the distribution of individual protein foods in protein food group based on protein item cluster[1,2]

Protein Item Clusters	% of Food group	Ounce eq/day from each protein food group
Eggs	9.20	0.5060
High omega fish	2.78	0.1529
Low omega fish	6.52	0.3586
Nuts/seeds	12.86	0.7073
Poultry	29.34	1.6137
Red meat	37.29	2.0510
Soy	2.01	0.1106
**TOTAL**	**100.00**	**5.50**

[1] Table 4.1: USDA Food Patterns -- Item Clusters, Representative Foods, and Percent of Consumption; in 2020 Dietary Guidelines Advisory Committee and Food Pattern Modeling Team. 2020. Food Pattern Modeling: Ages 2 Years and Older. U.S. Department of Agriculture, Washington, D.C. (Available at https://www.dietaryguidelines.gov/sites/default/files/2020-07/FoodPatternModeling_Report_2YearsandOlder.pdf; accessed 23 May 2023)
[2] Protein Foods Subgroup and Item Clusters (Pages 37-41), Data for those 19-70 y (which covers those with 2,000 kcal/day recommendation)

Step 2. Determine the recommended distribution of Protein Food Subgroups in HDP[2]

Protein Food Subgroup	Ounce eq/week	Ounce eq/day
Red Meat	12.5	1.8
Poultry	10.5	1.5
Eggs	3	0.43
Seafood	8	1.14
Nuts seeds, soy	5	0.71
TOTAL PROTEIN FOODS	**39**	**5.58**

[2] Table D14.3. Comparison of food groups and subgroups between the 3 USDA Food Patterns at the 2,000-kcal level; in Dietary Guidelines Advisory Committee. 2020. Scientific Report of the 2020 Dietary Guidelines Advisory Committee: Advisory Report to the Secretary of Agriculture and the Secretary of Health and Human Services. U.S. Department of Agriculture, Agricultural Research Service, Washington DC. (Available at https://www.dietaryguidelines.gov/2020-advisory-committee-report; accessed 23 May 2023)

Table S1. Per Capita and Beef Consumer Usual Intakes of Beef Based on NHANES 2001 - 2018

	Per Capita Usual Intake of Beef Types, g/day (oz/day; subpopulation size (n))									
	2-18 y			19-59 y			60+ y			2+ y
	All	M	F	All	M	F	All	M	F	All
Total Beef	31.90 ± 0.92 g (1.13 ± 0.03 oz; n=10,913)	36.64 ± 1.26 g (1.29 ± 0.04 oz; n=5,483)	26.97 ± 0.97 g (0.95 ± 0.03 oz; n=5,430)	47.14 ± 1.09 g (1.66 ± 0.04 oz; n=13,203)	60.80 ± 1.66 g (2.14 ± 0.06 oz; n=6,514)	33.11 ± 1.05 g (1.17 ± 0.04 oz; n=6,689)	40.73 ± 1.16 g (1.44 ± 0.04 oz; n=6,563)	51.54 ± 2.08 g (1.82 ± 0.07 oz; n=3,275)	31.62 ± 1.27 g (1.12 ± 0.04 oz; n=3,288)	42.23 ± 0.86 g (1.49 ± 0.03 oz; n=30,679)
Fresh Lean Beef	22.87 ± 0.81 g (0.81 ± 0.03 oz; n=10,913)	26.17 ± 1.07 g (0.92 ± 0.04 oz; n=5,483)	19.51 ± 0.79 g (0.69 ± 0.03 oz; n=5,430)	38.20 ± 1.02 g (1.35 ± 0.04 oz; n=13,203)	49.02 ± 1.47 g (1.73 ± 0.05 oz; n=6,514)	27.05 ± 0.98 g (0.95 ± 0.03 oz; n=6,689)	32.01 ± 1.23 g (1.13 ± 0.04 oz; n=6,563)	40.33 ± 2.09 g (1.42 ± 0.07 oz; n=3,275)	25.08 ± 1.15 g (0.88 ± 0.04 oz; n=3,288)	33.36 ± 0.84 g (1.18 ± 0.03 oz; n=30,679)
Processed Beef	7.50 ± 0.42 g (0.26 ± 0.01 oz; n=10,913)	9.04 ± 0.58 g (0.32 ± 0.02 oz; n=5,483)	5.91 ± 0.46 g (0.21 ± 0.02 oz; n=5,430)	6.36 ± 0.25 g (0.22 ± 0.01 oz; n=13,203)	8.34 ± 0.47 g (0.29 ± 0.02 oz; n=6,514)	4.33 ± 0.27 g (0.15 ± 0.01 oz; n=6,689)	6.63 ± 0.46 g (0.23 ± 0.02 oz; n=6,563)	9.15 ± 0.70 g (0.32 ± 0.02 oz; n=3,275)	4.51 ± 0.47 g (0.16 ± 0.02 oz; n=3,288)	6.69 ± 0.21 g (0.24 ± 0.01 oz; n=30,679)
Ground Beef	15.97 ± 0.62 g (0.56 ± 0.02 oz; n=10,913)	18.26 ± 0.86 g (0.64 ± 0.03 oz; n=5,483)	13.64 ± 0.65 g (0.48 ± 0.02 oz; n=5,430)	22.70 ± 0.78 g (0.80 ± 0.03 oz; n=13,203)	28.55 ± 1.04 g (1.01 ± 0.04 oz; n=6,514)	16.65 ± 0.85 g (0.59 ± 0.03 oz; n=6,689)	17.13 ± 0.72 g (0.60 ± 0.03 oz; n=6,563)	20.00 ± 1.20 g (0.71 ± 0.04 oz; n=3,275)	14.62 ± 0.91 g (0.52 ± 0.03 oz; n=3,288)	19.97 ± 0.58 g (0.70 ± 0.02 oz; n=30,679)

Beef Consumer Usual Intake of Beef Types, g/day (oz/day; subpopulation size (n))										
	2-18 y			19-59 y			60+ y			2+ y
	All	M	F	All	M	F	All	M	F	All
Total Beef	61.99 ± 1.65 g (2.19 ± 0.06 oz; n=5,712)	68.40 ± 2.34 g (2.41 ± 0.08 oz; n=3,017)	54.51 ± 1.61 g (1.92 ± 0.06 oz; n=2,695)	91.76 ± 1.19 g (3.24 ± 0.04 oz; n=6,762)	108.95 ± 1.86 g (2.41 ± 0.08 oz; n=3,639)	70.20 ± 1.49 g (2.48 ± 0.05 oz; n=3,123)	84.10 ± 1.61 g (2.97 ± 0.06 oz; n=2,975)	98.25 ± 3.05 g (3.47 ± 0.11 oz; n=1,591)	70.40 ± 1.92 g (2.48 ± 0.07 oz; n=1,384)	83.16 ± 0.94 g (2.93 ± 0.03 oz; n=15,449)
Fresh Lean Beef	64.23 ± 1.77 g (2.27 ± 0.06 oz; n=3,928)	71.20 ± 2.75 g (2.51 ± 0.10 oz; n=2,066)	55.92 ± 1.85 g (1.97 ± 0.07 oz; n=1,862)	91.21 ± 1.41 g (3.22 ± 0.05 oz; n=5,551)	107.43 ± 2.04 g (3.79 ± 0.07 oz; n=2,994)	70.61 ± 1.54 g (2.49 ± 0.05 oz; n=2,557)	80.85 ± 1.90 g (2.85 ± 0.07 oz; n=2,397)	95.23 ± 3.24 g (3.36 ± 0.11 oz; n=1,269)	67.32 ± 2.11 g (2.37 ± 0.07 oz; n=1,128)	83.37 ± 1.04 g (2.94 ± 0.04 oz; n=11,876)
Processed Beef	30.83 ± 1.38 g (1.09 ± 0.05 oz; n=2,710)	33.90 ± 1.65 g (1.20 ± 0.06 oz; n=1,481)	26.64 ± 1.74 g (0.94 ± 0.06 oz; n=1,229)	38.77 ± 1.63 g (1.37 ± 0.06 oz; n=2,129)	45.00 ± 2.22 g (1.59 ± 0.08 oz; n=1,216)	30.44 ± 1.69 g (1.07 ± 0.06 oz; n=913)	48.09 ± 2.94 g (1.70 ± 0.10 oz; n=888)	56.18 ± 3.61 g (1.98 ± 0.13 oz; n=503)	38.25 ± 3.55 g (1.35 ± 0.13 oz; n=385)	37.71 ± 1.22 g (1.33 ± 0.04 oz; n=5,727)
Ground Beef	57.29 ± 1.48 g (2.02 ± 0.05 oz; n=3,034)	62.90 ± 2.42 g (2.22 ± 0.09 oz; n=1,615)	50.53 ± 1.76 g (1.78 ± 0.06 oz; n=1,419)	76.59 ± 2.38 g (2.70 ± 0.08 oz; n=3,751)	89.51 ± 3.89 g (3.16 ± 0.14 oz; n=2,008)	61.11 ± 1.93 g (2.16 ± 0.07 oz; n=1,743)	71.49 ± 2.98 g (2.52 ± 0.10 oz; n=1,448)	81.68 ± 4.71 g (2.88 ± 0.17 oz; n=778)	61.94 ± 2.83 g (2.18 ± 0.10 oz; n=670)	71.16 ± 1.79 g (2.51 ± 0.06 oz; n=8,233)

Table S2. Day 1 Mean Intake of Total Ground Beef and Ground Beef From Fast Food Sources By Americans, Gender Combined – NHANES 2011-2018.

	**Per Capita Intake, g/day (oz/day; subpopulation size (n))**		**Beef Consumer Intake, g/day (oz/day; subpopulation size (n))**	
	Ground Beef	Ground Beef from Fast Food	Ground Beef	Ground Beef from Fast Food
2-18 years	16.1 ± 0.7 g (0.6 ± 0.02 oz; n=10,913)	4.6 ± 0.3 g (0.2 ± 0.01 oz; n=10,913)	56.98 ± 1.5 g (2.0 ± 0.05 oz; n=3,034)	60.5 ± 2.5 g (2.1 ± 0.1 oz; n=930)
19-59 years	22.9 ± 0.8 g (0.8 ± 0.03 oz; n=13,203)	8.05 ± 0.43 g (0.3 ± 0.02 oz; n=13,202)	76.9 ± 1.5 g (2.7 ± 0.1 oz; n=3,751)	74.4 ± 2.0 g (2.6 ± 0.1 oz; n=1,463)
60+ years	17.08 ± 1.01 g (0.6 ± 0.04 oz; n=6,563)	3.76 ± 0.35 g (0.1 ± 0.01 oz; n=6,559)	70.2 ± 2.4 g (2.5 ± 0.1 oz; n=1,448)	65.6 ± 4.2 g (2.3 ± 0.2 oz; n=404)
